# Use of Amino Acids as Supplements for Matching Nutrition, Training, and Rehabilitation—Focusing on Some Questions

**DOI:** 10.3390/nu17162667

**Published:** 2025-08-18

**Authors:** Francesco Saverio Dioaguardi

**Affiliations:** Dipartimento di Scienze Cliniche e di Comunità (DISCCO), University of Milan, 20122 Milano, Italy; fsdioguardi@gmail.com

**Keywords:** nutrition, essential amino acids, training, rehabilitation, aging

## Abstract

Exercise for improving physical performance or for rehabilitation and exercise for recovering from altered to normal efficiency of muscles, should be matched by adequacy of nutrition, thus supporting increased metabolic needs. Exercise triggers production of energy, and this is followed also by increased production of oxidant molecules, therefore, epigenetic adaptation in cells requires complex modifications, driven by an optimal balance between syntheses and autophagy, maintaining integrity and promoting increased efficiency of cells. Mitochondrial biogenesis, energy production, anti-oxidants systems, protein syntheses, and removal of inefficient structures are all subordinate to the sufficient availability of essential amino acids and they are indispensable to promote, activate, and maintain protein syntheses of all the structures, either contractile proteins or organelles, and related enzymatic systems on which physical efficiency is based. Increased needs or insufficient availability of essential amino acids in specific populations, due to peculiar changes in metabolism behaviors, as described in the training elderly, should be prevented and treated. Also, we are starting to understand the complexity of interactions among nutritional and physically driven activation of protein syntheses, and why only nutritional stimuli are poorly efficient in promoting muscle trophism even if essential amino acids remain indispensable for triggering syntheses. Moreover, the relationship between increased nutritional need and efficiency of immune system consequent to regular training should not be forgotten, particularly in rehabilitation programs for the elderly.

## 1. Introduction

Any type of training is targeted at ameliorating performances. Both training to perform better or to recover from illness, which is rehabilitation, increase nutritional needs. Not only do muscle fibers consume energy proportionally to loads when contracting [1], but a lot of energy is also spent on the synthesis of proteins. Thus, abundant availability of essential (E) amino acids (AAs) [2] and oxygen suitable for ATP production are both needed for protein syntheses. The evidence arouses the following questions: How increased production of ATP is supported? Which are the consequences of increasing oxygen consumption and ATP production?

## 2. Exercise Increases Nutritional Needs

Most responsive ATP production is dependent on mitochondria efficiency, and this requires activation of mitochondrial biogenesis, and, also, an increased supply of oxygen. Interestingly, it was shown that sensing abundant EAA availability activates not only protein syntheses [2], but also a coordinated sequence of epigenetic adaptations: increased activity of both endothelial nitrogen oxide synthases (eNOs) that improves oxygen availability and mitochondrial biogenesis through the activation of the SIRT1/PGC1α [3] (Silent Information Regulator homologs of the Sir 2, *S. cerevisiae*, gene protein 1/Peroxisome Proliferator-Activated Receptor Gamma Coactivator 1 alpha) axis [4]. Energy production by mitochondria controls the synthesis of proteins since any peptide bond requires about four ATPs (Adenosine Tri-Phosphates) to be completed [5], but, also, the production of ATPs by oxidation creates large amounts of reactive oxygen species (ROS). It has been experimentally shown that long-term abundant availability of EAAs, the molecules indispensable for protein syntheses, also epigenetically activates mitochondrial biogenesis and production and consumption of ATP and ROS production. In turn, these are intertwined with increased local expression and thus synthesis and activity of anti-oxidants enzymatic systems, such as gluthatione (GSH) and super-oxide dismutase (SOD) [6,7].

Thus, building muscle mass is not only expensive, but also produces enormous amounts of ROS, and this may seem paradoxical since exercise is predicted to prolong life while ROS are too often depicted as the worst enemies of cell integrities, and unmatched production of ROS by anti-oxidant systems have been implicated in the development of sarcopenia and blunted new mitochondria biogenesis [8].

The most important consideration is that ROS have a metabolic role, since signaling increased oxidative processes which are also efficient activators of autophagy [9] and are thus indispensable messengers to match the ATP driven synthesis of new structures and the recycling of aged and less efficient ones [10]. In conditions of poor nutritional status, EAAs are scarce negatively influencing syntheses and renewals of contractile proteins, and thus also renewal and maintenance of anti-oxidant enzymatic systems would be poor both due to precursor deficits but also to the reduced ATP syntheses and autophagy. In those conditions [7,8] epigenetic activation of mitochondrial biogenesis is suppressed as well as mitochondrial autophagy [11]. On the contrary, ROS-dependent autophagy for recovering from most serious damage and most efficient tissue remodeling has been shown to be indispensable even in the heart for repairing, continuous remodeling, and most efficient recovery after infarction [9]. But ATP concentration and consumption also control directly the balance between protein synthesis and autophagy, since the main gauge triggering autophagy, Adenosine Mono-Phosphate (AMP) kinase (K) which is activated by risen AMP concentrations following ATP utilization, also inhibits mTORC1-dependent protein syntheses.

**Figure 1:**
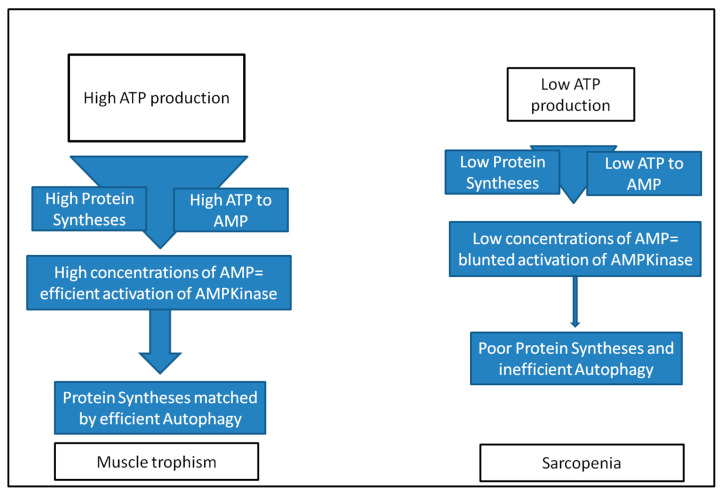
In Figure 1 is schematically resumed how changes in ATP production may influence not only protein syntheses, but also autophagy. At high ATP concentrations syntheses may proceed, and consumption of ATP to AMP would be proportionally elevated, and finally AMP concentration would rise at levels triggering AMPK activation driven efficient autophagy. On the contrary, when ATP production would be low, also protein syntheses would be blunted by scarce ATP availability, accordingly, AMP concentrations would poorly activate AMPK consequently not eliciting efficient autophagy.

Therefore, the most elevated would be ATP consumption due to protein syntheses, the proportional AMP concentrations would increase, and the activation of AMPK dependent autophagy would be triggered. A model explaining how high or low ATP production would influence positively or negatively both protein synthesis and autophagy has been recently suggested [12], and is resumed in Figure 1.

Figure 1 schematically presents how changes in ATP production may influence not only protein syntheses, but also autophagy. At high ATP concentrations syntheses may proceed, consumption of ATP to AMP would be proportionally elevated, and finally AMP concentration would rise to levels triggering AMPK activation-driven efficient autophagy. On the contrary when ATP production would be low, protein syntheses would be blunted by scarce ATP availability, accordingly, and AMP concentrations would poorly activate AMPK and so consequently efficient autophagy would not be elicited.

## 3. Digestion and Absorption of Proteins: Nutrition Training and Aging

Absorption of dietary proteins is negatively modified by aging and reduced absorption increases fecal content of proteins [13]. On the contrary, free amino acids are effectively absorbed in the upper intestinal tract without depending on most efficient digestion, and so there is a neat reduction in substrates potentially generating metabolic toxins by microbiota, such as those derived from bacterial metabolism of tryptophan contained both in indigested fractions of dietary proteins and in proteins secreted by the intestine [14], which have been particularly well studied [15]. Moreover, insufficient intake of amino acids in animals has been shown to further alter the efficiency of absorption of amino acids by proteins and the general efficiency of nutrition [16]. These facts pose several questions. Of interest, most people who train and most people requiring physical efforts for rehabilitation need to reduce their body weight or maintain weight without impairment of protein syntheses. This requires careful control of caloric intake but the maintenance or increase of nitrogen intake necessary to maintain, build, and renew the contractile proteins of muscle cells both in peripheral muscles and in the heart. Foods providing proteins may have different caloric contents as the percentage of protein compared to the total food weight is quite low, at best below 30%. This has led to the wide use of purified proteins, whose digestibility and quality based on amino acid absorption has been well studied [17]. Free amino acids provided by supplements have the advantage of having fewer calories than purified proteins and not needing to undergo digestion and so having maximal absorption; thus when free amino acids are used as a diet supplement they can overcome the reduced absorption of dietary proteins that comes with age [13]. Moreover, specific compositions may be formulated to fully meet human needs [18]. Benefits of supplementation by free amino acids in heart and muscular performance of patients affected by chronic heart failure have been clinically observed [19]. Also, a question often causing some concern is the circadian distribution of nitrogen intake. In the author’s opinion, multiple separated oral intakes are more efficient than one unique dose, as shown in studies in animals [20] and humans [21]. Indeed, there is still much to learn about the relationship between amino acid intakes and plasma patterns after oral loads, since both peak and time-dependent elevations in plasma concentrations are functions of the loaded amounts. Most elevated doses are followed by most elevated and prolonged plasma concentrations and effects on muscle protein synthesis. Smaller doses of amino acids are followed by lower and shorter peaks [22]. Of note, 20 g of just three amino acids caused plasma concentrations to significantly and constantly increase after 10 to 120 min [23]. These observations may be of interest while facing the reduced synthetic response to amino acids in older men [24], since amino acids stimulate activation of mTORC1 signaling and protein syntheses more efficiently than resistance exercise in young men [25]. Thus, to improve synthetic balance in muscle and overcome the anabolic inflexibility observed in the elderly [24], a possible strategy would not be to increase the amount of nitrogen per serving but to increase the number of servings in a day.

## 4. Nutrition and Exercise: Molecular Bases of Synergy

Adequacy of nitrogen introduction by food is an indispensable mediator for protein synthesis, providing energy, materials, and information to cells. Amino acids are efficient substrates for energy production, building blocks indispensable for protein syntheses, and activate those by specific sensing effectors [26,27]. Amino acids have a primary role and specific sense in activating mTORC1 and triggering protein synthesis [28]. So, why is nutrition indispensable but not sufficient to promote muscle growth and only partially efficient in reducing, but not abolishing [29], muscle loss after bed rest [30]? There is no exhaustive answer to this question, but the full picture about how mTORC1 is activated is too often ignored. Indeed, among the multiple negative inhibitors of mTORC1 that should be inactivated to trigger mTORC1 activity, Deptor (disheveled EGL-10 and pleckstrin domain-containing mTOR) has been identified, but its role underevaluated. DEPTOR brakes mTORC1 activation through RAPTOR very efficiently, being at the end of the CASTOR/SESTRIN/GATORs/RAGs cascade specifically activated by amino acids, particularly leucine and arginine [31]. Of note, DEPTOR should be inactivated by phosphorylation and thus mTORC1 activity can be finally triggered, and this should be obtained primarily by exercise [32]. These findings support the concept that both exercise and nutrition tightly control epigenetic factors at different levels, and all those should be considered strictly intertwined when considering medical interventions. Therefore, the best matching of timing and doses of exercise and nutrition are worth of careful and detailed studies [33,34].

## 5. Protein Malnutrition Compromises Immunity

It is well known that protein malnutrition compromises immune efficiency in humans [35], but despite its clinical relevance, its prevalence in a primary care setting is poorly documented [36]. On the contrary, significant data provided by the U.S. Army Research Institute of Environmental Medicine [37] show that protein–calorie restriction or insufficient introduction linked to logistical constraints and prolonged physical efforts may often be present in both men and women during training [38]. A major problem is that there are no established methods to effectively diagnose precocious malnutrition-dependent loss of immune function or to diagnose initial functional protein malnutrition in metabolically and physically very efficient individuals, although some biochemical parameters of malnutrition would be worth more attention and study [39]. There is evidence that amino acids are necessary for the integrity of immune function [40], and the recent COVID-19 pandemic revealed that protein malnutrition increases mortality risks at any age [41]. On the contrary, the preventive efficacy of chronic supplementation with EAAs was shown through effects on the immune system, even in the elderly, with both a reduced risk of infections by preventing malnutrition and the most efficient therapeutic response to antibiotic therapies in the case of long-term-care-acquired infections [42]. Thus, adequate monitoring and eventual supplementation of free amino acids and/or proteins should be regularly suggested to both young and elderly populations training at any intensity both to maximize efficiency of exercise [43], particularly in individuals considered at potential risk of protein insufficient introduction [44,45]. A most puzzling question is what ratios among EAAs and not-essential AAs (NEAAs) are most favorable, and also what are definitely the maximal unfavorable percentages of NEAAs provided by diets tolerable by human health. This question has been faced only in experimental settings, and studies showed that relatively small modifications, a 15% increase in NEAA percentages, was sufficient for reducing life-span in rodents [46].

## 6. Conclusions

Nitrogen provided by diet should match the increased energy requirements accompanying training. This is important whether training is aimed at improving performance or recovering from illness. Aging is a further challenge for training people, and benefits of exercise loads and quality of nutrition should be monitored particularly carefully in this population. Still, exercise is an irreplaceable practice for maintenance of healthy aging and constant physical training should be implemented at all ages. But, either quantity, quality, or distribution in time of nutritional support and eventually supplementation by proteins or also by EAAs to training people is worth being studied and understood more systematically.
